# Applications of Accelerometers and Other Bio-Logging Devices in Captive and Wild Animals

**DOI:** 10.3390/ani13020222

**Published:** 2023-01-06

**Authors:** Marco Campera, Marianna Chimienti, K. A. I. Nekaris

**Affiliations:** 1 Department of Biological and Medical Sciences, Oxford Brookes University, Oxford OX3 0BP, UK; 2 Centre d’Etudes Biologiques de Chizé, 405 Route de Prissé la Charrière, 79360 Villiers-en-Bois, France; 3 Nocturnal Primate Research Group, School of Social Sciences, Oxford Brookes University, Oxford OX3 0BP, UK

## Introduction to the Special Issue

Bio-logging devices have been widely used in ecology across a range of species to acquire information on the secret lives of animals in the wild, which would otherwise be challenging to obtain via direct observations [1]. Data obtained from bio-logging devices on animals both in captivity and in the wild have been used to assess several aspects of their biology and physiology, with different applications including estimating activity patterns, habitat use, energy expenditure, body temperature, and sleep, as well as mortality and reproductive events [2,3,4,5,6,7]. Devices have also rapidly developed in recent years in terms of reduced size, increased battery life, the number of sensors included, and the type of data that can be concurrently recorded [8]. Tiny devices now allow extensive data collection even on small animals.

While the applications of bio-logging have grown in ecology, its use in management and industrial applications allows novel information on animals’ ecology, physiology, and health status to be gained. In our Special Issue, we collected papers that investigated the use of accelerometers and other remote tracking systems in captive and wild animals, as well as farm and game animals with potential applications for wild animals. Eager et al. [9] developed a system (i.e., the IsoLynx system) to track greyhounds during races and reduce their race-related injuries. Similar techniques (i.e., video- or image-based animal tracking) have been used to track wild animals [10,11,12,13,14]. Nekaris et al. [15] used triaxial accelerometers to predict the behaviors of a captive individual of a nocturnal primate, the Bengal slow loris, via a random forest model. Triaxial accelerometers work by measuring and storing raw acceleration along three axes. From raw acceleration data, it is possible to calculate variables which can help us understand how animals move, such as static and dynamic acceleration, as well as the amplitude of dynamic acceleration, body pitch (vertical orientation of equipped animal), standard error, and overall dynamic body acceleration [2]. They estimated an accuracy of 80.7 ± SD 9.9% in predicting behaviors, with resting predicted with a 99.8% accuracy and a lower accuracy for feeding and locomotor behaviors. They highlighted the importance of captive settings for validating techniques to be implemented in the wild. Similarly, Pavese et al. [16] used triaxial accelerometers to predict behaviors of lesser anteaters in captivity. Both studies used video recording to calibrate accelerometer data. Jeantet et al. [17] used a convolutional neural network to identify the egg-laying process in sea turtles, with the potential to monitor nesting sea turtle populations with automated methods. Fischer et al. [18] described a novel mobile pressure sensor system for detailed gait analysis in dairy cows, with the consequent application for monitoring health in cows and other farm animals.

From our Special Issue, we highlighted the potential applications of bio-loggers to monitor health and injuries, predict animal behaviors, and monitor population growth. Importantly, we collected examples of knowledge acquired from either wild, captive, or farmed animals that can be shared across research, management, and industrial fields. These papers will contribute to the current knowledge base on bio-loggers and their potential for health and population monitoring in wild and captive animals, as well as in animals used by humans in different contexts.
